# Pulmonary Manifestations of Drug Reaction with Eosinophilia and Systemic Symptoms (DRESS) Syndrome: A Systematic Review

**DOI:** 10.1155/2019/7863815

**Published:** 2019-09-24

**Authors:** Pahnwat Tonya Taweesedt, Charles W. Nordstrom, Jessica Stoeckel, Igor Dumic

**Affiliations:** ^1^Icahn School of Medicine at Mount Sinai, New York, NY, USA; ^2^Mayo Clinic Health System, Eau Claire, WI, USA; ^3^Mayo Clinic Alix College of Medicine and Science, Rochester, MN, USA; ^4^Albert Einstein College of Medicine, Bronx, NY, USA

## Abstract

**Background:**

The syndrome of drug reaction with eosinophilia and systemic symptoms (DRESS) is a rare, yet potentially fatal hypersensitivity reaction, most commonly associated with anticonvulsants, sulfonamides, and allopurinol. The reaction commonly manifests as a febrile skin eruption with lymphadenopathy and malaise between two and eight weeks following drug exposure. Internal organ involvement occurs in close to 90 percent of patients, and multiple organs may be involved in approximately half of those affected (most commonly the liver, kidney, and lung). Its long latency period and its variable clinical pattern of presentation have earned it the moniker of “the great mimicker,” with delays in diagnosis leading to higher morbidity and mortality. Although less commonly affected in DRESS syndrome, lung involvement is associated with more severe clinical course and potentially worse outcome. Pulmonary symptoms may precede development of the other more common symptoms and signs of the syndrome, or they might develop later in the course of the disease. Lung involvement in DRESS presents with a plethora of manifestations from mild cough or dyspnea with nonspecific interstitial changes on chest imaging to acute respiratory distress syndrome (ARDS) with life-threatening hypoxic respiratory failure.

**Methods:**

We performed a systematic review of literature from the PubMed database and selected cases of definite DRESS syndrome as defined by the European Registry of Severe Cutaneous Adverse Reactions (RegiSCAR) with a score of 6 or more who also had pulmonary involvement. Demographic data, pattern of lung involvement, culprit medication, latency period, laboratory findings, therapy, and outcome were described and compared with the literature.

**Results:**

The most common pulmonary radiographic findings in DRESS were interstitial infiltrates in 50% of cases, followed by acute respiratory distress syndrome (ARDS) 31%. Symptoms of cough and shortness of breath (SOB) were present in 72% of patients at the time of presentation. SOB was the more common presenting symptom (81%) compared to cough (19%). In 95% of cases, another visceral organ was involved (most commonly liver or kidneys). 45% of cases were initially misdiagnosed as pneumonia and were treated with empiric antimicrobials. In a multivariate regression, a latency of 30 days or less and an age of 60 or less were associated with development of ARDS. Gender and eosinophil count were not associated with severity of pulmonary manifestations. All patients recovered, and in the vast majority of cases (95%), parenteral steroids were used for treatment in addition to supportive care and symptomatic management.

**Conclusion:**

Albeit rare, DRESS is a potentially life-threatening syndrome which may present with a myriad of pulmonary signs and symptoms. Pulmonary manifestations are less common but are typically seen in more severe cases. Pulmonary manifestations may be a presenting sign of DRESS, and timely recognition is important in order to stop offending medication and decrease morbidity and mortality.

## 1. Introduction

Drug reaction with eosinophilia and systemic symptoms (DRESS) syndrome, formerly known as drug-induced hypersensitivity syndrome (DIHS), is an acute, idiosyncratic, and potentially life-threatening drug reaction. DRESS is characterized by combinations of the following: fever higher than 38.5°C, skin eruptions, hematologic abnormalities (most commonly eosinophilia), lymphadenopathy, and variable visceral organ involvement [1]. The most commonly implicated medications are antimicrobials, anticonvulsants, and allopurinol. Its prevalence ranges from 1 in 1,000 to 1 in 10,000 with mortality as high as 10%. While the liver is the most commonly affected visceral organ in DRESS, other internal organs can be affected as well including the kidneys, lung, intestines, pancreas, thyroid, heart, and brain [2]. Mortality in DRESS syndrome is linked to the extent of internal organ involvement with death usually resulting from myocarditis, liver failure, or respiratory failure. Since there is no specific test to accurately diagnose DRESS syndrome, diagnosis depends on a high index of suspicion, history of relatively recent exposure to the drug, and by excluding other common mimickers of DRESS such as infection, inflammation, neoplastic diseases, and other cutaneous reactions which can present in similar fashion [1, 2].

Lungs are less frequently involved in DRESS syndrome, but their involvement may be associated with a more severe clinical course and higher mortality. Pulmonary manifestations in DRESS are variable and may include nonspecific interstitial pneumonitis, pleural effusion, pneumonia, pulmonary nodules, and (in the most severe cases) acute respiratory distress syndrome (ARDS). Patients with pulmonary manifestations commonly present with dyspnea, cough, and/or pleurisy.

In order to better describe the various pulmonary manifestations, their potential relationship to classes of medication, and their specific outcomes, we performed a systematic review of the literature on this topic. To the best of our knowledge, this is the first systematic review of the literature specifically describing pulmonary manifestation in DRESS syndrome.

## 2. Materials and Methods

### 2.1. Database and the Key Words (MeSH)

A systematic review of the literature was performed by using PubMed database for case reports and case series of DRESS syndrome with lung involvement from the database inception to May 2019.

The following key words alone and/or in combination were used: “DRESS and lung,” “DRESS and pneumonia,” “DRESS and Pneumonitis,” “DRESS and Pleural effusion,” “DRESS and ARDS,” “DIHS and lung,” “DIHS and Pneumonia,” “DIHS and Pneumonitis,” “DIHS and pleural effusion,” and “DIHS and ARDS.”

### 2.2. Definitions

Definite cases were defined as cases that had a score of at least 6 or more in accordance with the RegiSCAR (European Registry of Severe Cutaneous Adverse Reactions) scoring system. RegiSCAR was developed to more accurately define different entities among febrile skin eruptions (such as DRESS, Steven–Johnson syndrome, acute generalized exanthematous pustulosis, and toxic epidermal necrolysis among the others) (Table 1) [3].

Pulmonary involvement was defined by symptoms or/and radiological findings. Symptoms included cough, dyspnea, and/or pleurisy. Radiological findings included unilateral or bilateral interstitial infiltrates, pleural effusion, lobar infiltrate, and/or pulmonary nodules.

### 2.3. Selection Criteria

We selected only definite cases with pulmonary involvement. Duplicate articles, articles in languages other than English, narrative reviews, and cases of DRESS syndrome without lung involvement were excluded.

**Figure 1:**
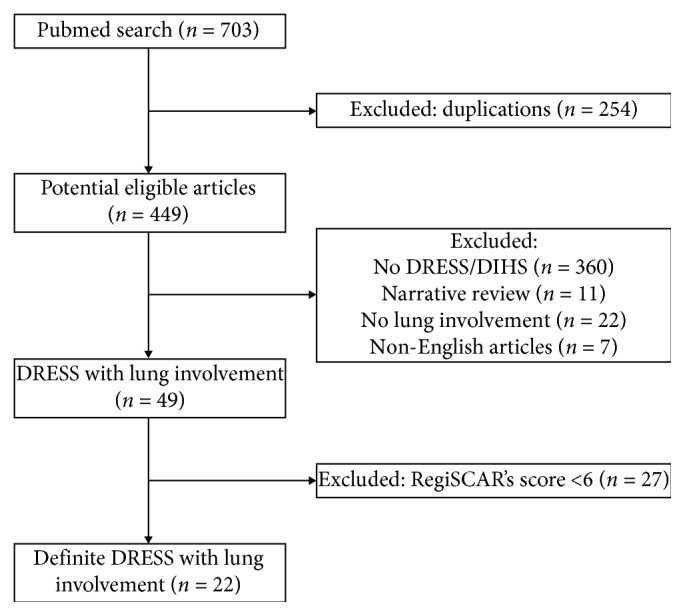
Flow chart of methodology and literature selection process.

The flow chart of selection of the final cases included in analysis is illustrated in Figure 1.

### 2.4. Data Collection

Two researchers independently and blindly identified and selected the titles, abstracts, and full texts obtained in the database search. Discrepancies of the selected articles were resolved by consensus. Subsequently, we screened the reference list of selected articles to identify any further articles for inclusion, in accordance with the selection criteria. An Excel table was constructed, and for each case, we collected patients' demographic data, comorbid conditions, type of lung involvement, severity of eosinophilia, other visceral organs involved, offending medication, latency period, results of the tissue biopsy (if done), treatment of each case, and outcomes (Table 2).

### 2.5. Statistical Analysis

The program Stata/MP 14.2 was used for statistical analysis. Continuous data were reported as mean. Categorical data were presented as percentage. The multivariate regression model was used to examine factors associated with lung manifestation in DRESS syndrome. *p* value <0.05 was defined as statistically significant.

## 3. Results and Discussion

### 3.1. Demographic Characteristics of Patients with DRESS Syndrome with Pulmonary Involvement

In published cases to date, the age of patients ranged from 4 to 77 years (mean 34 years) [4–25]. While extremes in age are a recognized risk factor in conditions like drug-induced interstitial lung disease (DILD), we did not observe any age predilection in DRESS. One potential explanation for this difference might be that while DRESS is idiosyncratic, other drug-induced toxicities are dose dependent. The prevalence of DRESS is higher in adults than in children; however, children can be equally severely affected, as illustrated in the two case reports by Castellazzi et al. which exemplify the importance of considering DRESS in the pediatric population as well [26]. While there was no clear age predilection in DRESS, severity of pulmonary manifestation appears to be associated with age, and in multivariate analysis, an age of 60 or less was associated with development of ARDS (Table 3).

In this review of 22 cases, 13 cases (59%) were described in men; however, gender has not been identified as a risk factor in development of DRESS syndrome and is not associated with severity of pulmonary manifestations. This contrasts with conditions like nitrofurantoin-induced chronic lung toxicity which is more commonly seen in females [27]. While age and gender demonstrate no correlation to development of DRESS, there does appear to be an association with race. This correlation appears to be dependent on specific human leucocyte antigen (HLA) alleles which vary among different ethnicities. For example, minocycline, which is commonly associated with pulmonary involvement in DRESS syndrome, seems to be more prevalent among Caribbean blacks. Additionally, HLA-B*∗*5701, HLA-B*∗*5801, HLA-B*∗*56:02, and HLA-A*∗*31:01 are, respectively, associated with higher prevalence of abacavir-induced hypersensitivity (more common in whites); allopurinol-induced DRESS (particularly prevalent among specific Chinese groups); phenytoin-induced DRESS (particularly prevalent in Aboriginal Australians); and carbamazepine-induced hypersensitivity reaction (among both Northern and Southern European populations) [28–31]. While HLA-A*∗*31:01 is a risk factor for development of all severe cutaneous reactions to carbamazepine, its association with DRESS syndrome appears to be the strongest [31]. In our review, however, race was not described in many cases; therefore, it was not included in our analysis.

Presence of comorbidities and preexisting pulmonary disease might be a risk factor for development of DRESS with pulmonary involvement; however, there is no strong evidence to support this statement, and this question remains a subject of ongoing research. In our review, the majority of patients had comorbidities, of which some were the indication for prescribing the culprit medication. Interestingly, in our review, only 3 patients had underlying chronic disease related to lung pathology (pulmonary TB), while the majority of cases (19 of 22) had no preexisting lung disease. Unfortunately, smoking habits were not reported in the vast majority of patients (95%), so we are unable to comment on possible association between smoking and pulmonary manifestations of DRESS.

### 3.2. Extent of Visceral Organ Involvement in Patients with DRESS Syndrome Who Had Pulmonary Manifestations

In addition to pulmonary involvement, 81% of cases (18 of 22) had hepatic involvement, 36% (8 of 22) had renal involvement, and 13.5% (3 of 22) had brain involvement with encephalopathy and seizures. In 31% of reviewed cases (7 of 22), more than 3 visceral organs were involved, with the spleen, colon, and heart being affected in one case each. Only one case [8] had 4 visceral organs involved manifesting with nephritis, colitis, pneumonitis, and potential cardiac involvement in the form of atrial fibrillation. Interestingly, in that particular case, despite multiorgan involvement, there was no reported hepatic disease.

Prior retrospective studies have shown that the liver is the most commonly affected organ in DRESS such as those conducted by Lee et al. [32] and Chen et al. [33]. In these two retrospective studies which included 25 and 60 DRESS patients, respectively, both described liver involvement in 80%, followed by renal involvement (28% and 40%, respectively) and pulmonary involvement (20% and 33% respectively). Interestingly, a systematic review of the literature by Cacoub et al. [2] found that lung involvement was significantly less common and was identified in only 5% of reported cases. Alternatively, a retrospective study of 15 patients treated in the ICU in France over the period of 12 years demonstrated that 11 out of 15 patients (73.3%) had severe lung involvement. The authors of that study suggested that lung involvement might be associated with a worse clinical course and outcome since mortality in this retrospective analysis was 20%, with 3 of 15 patients dying [34]. Given the rarity of DRESS syndrome and particularly those cases with lung involvement, the exact prevalence remains unknown.

Extrahepatic gastrointestinal involvement is comparatively rare in DRESS although colitis and pancreatitis have been described [8]. It may be that gastrointestinal manifestations are underreported since they generally tend to be nonspecific and mild.

The endocrine system is commonly spared in the acute phase of DRESS syndrome, but post-DRESS diabetes mellitus type I and hypothyroidism have been described within a few months to years following DRESS syndrome. These manifestations do not appear to correlate to the class of culprit medication [35, 36].

### 3.3. Pattern of Lung Involvement in DRESS Syndrome

DRESS syndrome typically starts insidiously with fever. Visceral organ involvement may precede development of the more easily recognized cutaneous manifestations. Sometimes, the lungs are the first organ affected with patients mistakenly diagnosed with pneumonia (Table 2). Pulmonary involvement in DRESS commonly manifests symptomatically as dyspnea, cough, or pleurisy [37, 38]. A plethora of pulmonary findings have been described as part of DRESS syndrome including impaired pulmonary function tests (PFTs), interstitial pulmonary infiltrates, pneumonia, pulmonary nodules, pleural effusion, and, in the most severe cases ARDS with acute hypoxemic respiratory failure. While not classically described as pulmonary finding, mediastinal lymphadenopathy is another manifestation and may occur in the absence of peripheral lymphadenopathy [8].

In this systematic review, we found that the most common pulmonary manifestations were interstitial infiltrates diagnosed as pneumonitis in 11 of 22 cases (50%). ARDS was described in 7 of 22 cases (31%). Pleural effusion was the next most common radiological manifestation, described in 5 of 22 cases (22.7%). Lobar infiltrates and pulmonary nodules were described in 3 cases each. The study by Lee et al. [32] described 5 of 25 patients with pulmonary involvement (20% of the entire cohort), and of these patients, 3 had pulmonary infiltrates and 2 had pleural effusion as the pulmonary manifestation of DRESS syndrome.

The majority of reviewed cases (72%) had pulmonary symptoms on admission. Of these cases, 81% reported SOB, 19% reported a dry cough, and 19% reported both.

In the multivariate regression model, a latency period of less than 30 days and age of 60 years or less were associated with development of severe pulmonary manifestation of DRESS syndrome, i.e., ARDS (Table 3).

### 3.4. Differential Diagnosis of DRESS Syndrome with Pulmonary Involvement

The combination of peripheral eosinophilia, rash, and respiratory symptoms is seen in many conditions which can be divided into two categories: infectious and noninfectious.

Among noninfectious causes, most important to consider are as follows:

- Neoplastic (leukemia and lung cancer)
- Medications (non-DRESS-related reactions usually due to daptomycin or nitrofurantoin)
- Allergic (allergic bronchopulmonary aspergillosis)
- Autoimmune/inflammatory systemic conditions such as Churg-Strauss vasculitis, acute eosinophilic pneumonia, eosinophilic granulomatosis with polyangitis, idiopathic hyper-eosinophilic syndrome, and another “great mimicker”—systemic lupus erythematous

Infectious causes are numerous, and differential should include viral, bacterial, parasitic, and fungal infections.

If DRESS presents without eosinophilia and the dominant clinical features are febrile skin eruption with pulmonary infiltrates, then differential diagnosis should concentrate on viral exanthema or bacterial causes of community-acquired pneumonia that can present with rash in addition to typical pulmonary symptoms (e.g., *Mycoplasma pneumoniae*, *Chlamydia* spp., and secondary syphilis).

Endemic mycosis in the United States can present with eosinophilia and pulmonary infiltrates (e.g., *Coccidiomycosis*, *Paracoccydiomycosis*, *Histoplasmosis*, and *Cryptococcosis*). Parasitic infections are particularly important in the differential, especially in patients who have travelled to Asia and Middle Eastern countries where such infections are endemic. Transient pulmonary infiltrates accompanied by fever and peripheral eosinophilia are seen in both Katayama syndrome (acute schistosomiasis) and acute larva migrans (Loeffler's syndrome) due to infection from ascaris, hookworm, or strongyloides. The particularly high parasite burden associated with strongyloides hyperinfection syndrome can have predominant pulmonary manifestations and marked peripheral eosinophilia. Paragonomiasis, toxocara, and lymphatic filariasis (tropical pulmonary eosinophilia) are other parasites that can mimic DRESS syndrome in appropriate setting [39].

Differential diagnosis should be further expanded in immunocompromised patients such as in HIV-infected individuals. Such examples include raltegravir-induced DRESS with extensive pulmonary manifestations [19], opportunistic infections such as *Mycobacterium avium* complex or *Pneumocystis jiroveci*, AIDS-defining Kaposi sarcoma with lung involvement, and immune reconstitution syndrome (IRIS).

When there is other visceral organ involvement (as seen in 95% of cases when DRESS syndrome has pulmonary manifestations), additional specific syndromes and infections need to be considered. For example, if the kidneys are also involved, then pulmonary-renal syndrome (Goodpastures) should be considered. If there is hepatic involvement, then hepatopulmonary amebiasis should be ruled out (*Entamoeba histolytica*) [8, 39].

Clearly, the differential diagnosis of DRESS with pulmonary involvement is vast. Appropriate diagnosis and treatment requires an astute physician with a high index of suspicion, who can recognize the temporary association of symptoms with exposure to medications and rule out other “mimickers.” The diagnosis of DRESS syndrome rests on ruling out other potential etiologies in addition to being able to demonstrate exposure to medication in the recent past.

As demonstrated in Table 2, 10 of 22 patients (45%) were initially suspected to have pneumonia and were empirically treated with antimicrobials. These findings are in concordance with another retrospective study that demonstrated that 50% of patients with DRESS syndrome were initially misdiagnosed and treated for infection [40]. Ruling out infectious etiology is particularly important since corticosteroids which are the cornerstone of therapy for DRESS syndrome with visceral involvement might exacerbate particular infections and are relatively contraindicated. Another common misdiagnosis of patients with DRESS is lymphoma, for which is DRESS misdiagnosed in up to 75% of cases [2, 40]. This exemplifies the statement that clinicians rarely consider DRESS syndrome when evaluating febrile patients with rash and pulmonary involvement.

Bronchoscopy is not necessary for diagnosis of pulmonary involvement in DRESS syndrome but should be performed when there is a suspicion for eosinophilic lung disease. Unlike acute eosinophilic pneumonia (AEP) where there is an increase in percentage of eosinophils in broncoalveolar lavage (BAL) typically in the absence of peripheral eosinophilia, the hallmark of DRESS is an increased peripheral eosinophilia [41].

### 3.5. Implicated Medications and Latency

It has been recognized that organ involvement in DRESS syndrome can correlate with specific medications. It has been previously described that allopurinol is frequently associated with renal involvement in DRESS, while pulmonary manifestations often result from minocycline-induced DRESS [42]. While the list of medication that can cause DRESS syndrome is extensive, the list of medications that have been described to cause DRESS with predominantly pulmonary involvement is shorter (Table 2).

Minocycline and abacavir have been traditionally described to be more commonly associated with pulmonary manifestations in DRESS [37, 42, 43].

A particular association of minocycline-induced DRESS is the possibility of higher incidence of postsyndrome endocrinopathies such as hypothyroidism and type I DM [44]. Additionally, the majority of such cases have been reported in Japan which further emphasizes the possibility that genetics may predispose patients to react differently to a culprit medication. Similar to the cross reactivity among aromatic anticonvulsive medications, there is the possibility of cross reactivity among tetracyclines, as described by Robles et al. in case that involved severe DRESS with ARDS attributed to doxycycline [12].

Abacavir hypersensitivity has been well recognized and described. Due to possibility of life-threatening hypersensitivity reaction that can lead to circulatory shock, it is mandatory to test patients for HLA-B*∗*5701 prior to initiation of therapy. While this hypersensitivity primarily manifests as febrile rash and gastrointestinal symptoms, respiratory symptoms such as tachypnea, wheezing, and pharyngitis are not uncommon. These respiratory symptoms might be predominant and mimic upper respiratory infection. Patients with higher CD8 T-cell count and of white race are at higher risk to develop these reactions [45].

A relatively long latency from administration of medication to the onset of drug reaction is signature characteristics of DRESS syndrome. The latency period is usually 2–6 weeks but has been described to be as long as 105 days [46]. Shorter latency periods have been observed in the pediatric population, however, with one prospective study in children with DRESS due to antimicrobials exhibiting a latency period averaging 5.6 days [47]. The latency period in this review ranged from 2 to 72 days (median 29 days). In multivariate analysis, a latency period of less than 30 days was associated with ARDS development.

### 3.6. Treatment and Outcome

Treatment of DRESS syndrome is based on case reports and expert opinion. Due to the rarity of cases, there are no prospective studies that evaluate efficacy of different treatments [48]. The cornerstone of therapy is prompt recognition and withdrawal of the offending medication. Use of immunosuppressive medications is based on severity of symptoms and extent of visceral organ involvement. In mild cases, topical steroids and antihistamines are usually sufficient, while systemic steroids are needed for visceral organ involvement. In our review, all patients with pulmonary involvement but one (95%) received systemic immunosuppressive therapy. Of these, 18 received only steroids (81%) and 3 patients (14%) received a combination of intravenous immunoglobulins (IVIG) and steroids. The duration of therapy is usually prolonged, and a slow taper over several weeks is necessary to avoid recurrence which has been documented in cases with more abrupt withdrawal [2, 4, 48]. In cases with significant gastrointestinal involvement, oral route of steroids should be avoided due to unpredictable absorption.

## 4. Conclusion

Despite being rare, DRESS is a potential life-threatening syndrome which may present with myriad of pulmonary signs and symptoms. While pulmonary manifestations are less common, they are typically seen in more severe cases. Pulmonary manifestations may be a presenting sign of DRESS and are frequently misdiagnosed for pneumonia. Timely recognition is important in order to stop offending medication and improve the outcome.

Limitations of our systematic review are exclusion of cases that are published in languages other than English and those published in journals not indexed in PubMed.

## Figures and Tables

**Table 1:** DRESS validation score, known as RegiSCAR (adapted from Kardaun).

Score	−1	0	1	2	Min	Max
Fever ≥38.5°C	No/U	Yes			−1	0
Enlarged lymph nodes		No/U	Yes		0	1
Eosinophilia		No/U			0	2
Eosinophils			700–1499/*μ*l	≥1500/*μ*l		
Eosinophils, if leukocytes <4000			10–19.9%	≥20%		
Atypical lymphocytes		No/U	Yes		0	1
Skin involvement					−2	2
Skin rash extent (% BSA)		No/U	>50%			
Skin rash suggesting DRESS	No	U	Yes			
Biopsy suggesting DRESS	No	Yes/U				
Organ involvement^*∗*^					0	2
Liver		No/U	Yes			
Kidney		No/U	Yes			
Lung		No/U	Yes			
Muscle/heart		No/U	Yes			
Pancreas		No/U	Yes			
Other organ(s)		No/U	Yes			
Resolution ≥ 15 days	No/U	Yes			−1	0
Evaluation of other potential causes						
ANA						
Blood culture						
Serology for HVA/HVB/HVC/*Chlamydia*/*Mycoplasma pneumoniae*						
Other serology/PCR						
If none, positive, and ≥3 or above, negative			Yes		0	1
Total score					−4	9

BSA, body surface area; HAV, hepatitis A virus; HBV, hepatitis B virus; HCV, hepatitis C virus; DIHS, drug-induced hypersensitivity syndrome; DRESS, drug reaction/rash with eosinophilia and systemic symptoms; SCAR, severe cutaneous adverse reactions; U, unknown/unclassifiable. ^*∗*^After exclusion of other explanations: 1 = 1 organ; 2 = ≥2 organs. Final score <2: no case; final score 2-3: possible case; final score 4-5: probable case; final score >5: definite case.

**Table 2:** Case reports of definite DRESS syndrome with pulmonary involvement.

Reference	Age	Sex	Comorbidities	Smoking history	Lung involvement (radiologically)	Pulmonary symptoms present on admission	Empirically treated for PNA	Other visceral organs involved	Drugs	Onset (in days)	Eosinophil counts	EBV	HHV6	Treatment	Outcome
Hase et al. [4]	64	M	Diabetic neuropathy	NA	Interstitial infiltrates	Yes SOB	No	Liver	Carbamazepine	42	2192	Negative	Negative	Prednisolone and IVIG	Alive
Sawata et al. [5]	51	F	Crohn's disease	NA	Bilateral nodules	Yes SOB	No	Liver	Mesalazine and bactrim	72	5292	NA	Positive	Prednisone	Alive
Petkov et al. [6]	32	M	Epilepsy	NA	Interstitial infiltrates and ARDS	No (SOB later)	No	Liver	Lamotrigine	7	1530	NA	NA	Methylprednisolone	Alive
Karakaali et al. [7]	6	M	None	NA	Lobar infiltrate and pleural effusion	Yes Cough	Yes	Liver	Cefotaxime and clindamycin	11	430	Negative	NA	Methylprednisolone	Alive
James et al. [8]	63	M	HTN, spinal stenosis, and lower extremities edema	NA	Interstitial infiltrates	Yes Dry cough	Yes	Heart, kidney, and colon	Furosemide	70	1290	Negative	Negative	Prednisone	Alive
Shibuya et al. [9]	46	F	Subarachnoid hemorrhage and hereditary hemorrhagic telangiectasia	NA	Bilateral nodules	Yes Dry cough	No	Liver	Zonisamide	41	NA	Negative	Positive	Prednisone	Alive
Leão et al. [10]	77	F	HTN and DM	NA	Lobar infiltrate and pleural effusion	No (SOB later)	No	Kidney	Nitrofurantoin	3	8500	NA	NA	Prednisone	Alive
Hassan et al. [11]	73	M	HTN, HLD, CAD, and hyperuricemia	Heavy smoker	Interstitial infiltrate	Yes SOB and dry cough	Yes	Liver, kidney, and brain	Allopurinol	30	3408	Negative	NA	Methylprednisolone	Alive
Robles et al. [12]	20	F	Acne vulgaris	NA	ARDS	No (SOB later)	Yes	Liver and kidney	Doxycycline	21	980	Negative	NA	Methylprednisolone	Alive
Naniwa et al. [13]	61	M	Mumps, pulmonary tuberculosis, and systemic sclerosis	NA	Interstitial infiltrates	Yes SOB	No	None	Bactrim	21	10,032	Negative	Negative	Methylprednisolone	Alive
Gómez-Zorrilla et al. [14]	31	M	Astrocytoma	NA	Interstitial infiltrates	Yes SOB	No	Liver	Levetiracetam	46	NA	NA	NA	Dexamethasone	Alive
O'Meara et al. [15]	66	M	Hemochromatosis	NA	Interstitial infiltrates	Yes Dry cough and SOB	No	Liver, kidney, and brain	Vancomycin	28	3620	Negative	NA	Hydrocortisone	Alive
Aouam et al. [16]	14	M	Absence epilepsy	NA	Interstitial infiltrates	No	No	Liver	Carbamazepine and lamotrigine	48	3450	Negative	Negative	Discontinue medication and supportive care	Alive
Lee et al. [17]	29	F	Possible pulmonary TB	NA	Lobar infiltrate and pleural effusion	Yes SOB	No	Liver	Celecoxib and ethambutol	38	4477	Negative	Positive	Methylprednisolone and IVIG	Alive
Nawaz and Wall [18]	19	M	Spinal fracture	NA	Pleural effusion and interstitial infiltrates	Yes SOB	Yes	Liver and kidney	Titanium and minocycline	45	8704	NA	NA	Prednisone	Alive
Yee et al. [19]	18	F	HIV	NA	Lung nodules	No	Yes	Liver	Raltegravir	35	1300	Negative	NA	Prednisone	Alive
Clayton et al. [20]	26	M	Acne vulgaris	NA	Interstitial infiltrate and ARDS	No	Yes	Liver and brain	Minocycline	14	3250	NA	NA	IV corticosteroid	Alive
Bruwiere et al. [21]	35	F	Endocarditis	NA	ARDS	No	Yes	Liver and kidney	Teicoplanin, moxifloxacin, and ciprofloxacin	9	590	NA	NA	Prednisone	Alive
Wilcox et al. [22]	39	M	DM and HLD	NA	ARDS	Yes SOB	Yes	Kidney	Vancomycin	2	270	NA	NA	Methylprednisolone	Alive
Roca et al. [23]	22	F	Acne vulgaris	NA	ARDS	Yes Cough and SOB	Yes	Liver	Minocycline	2	5760	Negative	NA	High-dose steroids	Alive
Wang and Li [24]	50	M	Pulmonary tuberculosis	NA	Interstitial infiltrates and pulmonary effusion	No	No	Liver	Isoniazid, rifampicin, and pyrazinamide	16	2870	NA	NA	Prednisone and IVIG	Alive
Irga et al. [25]	4	F	Epilepsy	NA	ARDS	No	No	Liver and spleen	Carbamazepine	42	43,500	Negative	Positive	Methylprednisolone	Alive

M = male; F = female; HTN = hypertension; HLD = hyperlipidemia; NA = not applicable; IVIG = intravenous immunoglobulins; PNA = pneumonia; SOB = shortness of breath.

**Table 3:** Estimates from the linear probability model run via Stata/MP 14.2.

Explanatory Variables	Dep. var.: ARDS
Male	−0.0834
	(0.692)
Age below 60 years old	0.4217^*∗∗*^
	(0.035)
Absolute eosinophil count: 500–1500	0.2851
	(0.570)
Absolute eosinophil count: above 1500	0.1230
	(0.797)
Latency below 30 days	0.4845^*∗∗*^
	(0.013)
Intercept	Yes
Observations	22
*R*-squared	0.4576

*Note*. The values in parentheses denote *p* values: ^*∗∗∗*^*p* < 0.01; ^*∗∗*^*p* < 0.05; ^*∗*^*p* < 0.1.

## Supplementary Materials

Supplementary Materials The supplementary table summarizes case reports of definite DRESS syndrome with pulmonary involvement.
