# Supplementary material for: Pulmonary Manifestations of Drug Reaction with Eosinophilia and Systemic Symptoms (DRESS) Syndrome: A Systematic Review
Source: Biomed Res Int. 2019 Sep 24;2019:7863815. doi: 10.1155/2019/7863815 (PMC6778864; doi:10.1155/2019/7863815)
Supplement: Supplementary Materials — The supplementary table summarizes case reports of definite DRESS syndrome with pulmonary involvement. [file 7863815.f1.pdf]

Table S1. This table summarizes case reports of definite DRESS syndrome with pulmonary involvement.

Reference	Age (years)	Sex	Comorbidities	Smoking history	Lung involvement (radiologically)	Pulmonary symptoms present on admission	Empirically treated for PNA	Other visceral organs involved	Drugs	Onset (days)	Eosinophil counts (cells/μL)	EBV	HHV6	Treatment	Outcome
Hase I et al(4)	64	M	DM and diabetic neuropathy	NA	Interstitial infiltrates	Yes (SOB)	No	Liver	Carbamazepine	42	2192	Neg	Neg	Prednisolone IVIG	Alive
Sawata T et al(5)	51	F	Crohn's disease	NA	Bilateral nodules	Yes (SOB)	No	Liver	Mesalazine, Bactrim	72	5292	NA	Pod	Prednisone	Alive
Petkov T et al(6)	32	M	Epilepsy	NA	Interstitial infiltrates ARDS	No (SOB later)	No	Liver	Lamotrigine	7	1530	NA	NA	Methyl-prednisolone	Alive
Karakaali B et al (7)	6	M	None	NA	Lobar infiltrate Pleural effusion	Yes (Cough)	YES	Liver	Cefotaxime, Clindamycin	11	430	Neg	NA	Methyl-prednisolone	Alive
James J et al(8)	63	M	HTN, Spinal stenosis, Lower extremities edema	NA	Interstitial infiltrates	Yes (Dry Cough)	YES	Heart, Kidney Colon	Furosemide	70	1290	Neg	Neg	Prednisone	Alive
Shibuya R et al(9)	46	F	Subarachnoid hemorrhage, Hereditary Hemorrhagic telangiectasia	NA	Bilateral nodules	Yes (Dry cough)	No	Liver	Zonisamide	41	NA	Neg	Pos	Prednisone	Alive

Leão RN et al(10)	77	F	HTN and DM	NA	Lobar infiltrate Pleural effusion	No (SOB later)	No	Kidney	Nitrofurantoin	3	8500	NA	NA	Prednisone	Alive
Hassan S et al (11)	73	M	HTN, HLD, CAD, hyperuricemia	heavy smoker	Interstitial infiltrate	Yes (SOB, dry cough	YES	Liver Kidney Brain	Allopurinol	30	3408	Neg	NA	Methyl-prednisolone	Alive
Robles DT et al (12)	20	F	Acne vulgaris	NA	ARDS	No (SOB later)	YES	Liver, Kidney	Doxycycline	21	980	Neg	NA	Methyl-prednisolone	Alive
Naniwa T et al (13)	61	M	Mumps, pulmonary tuberculosis, systemic sclerosis	NA	Interstitial infiltrates	Yes SOB	No	None	Bactrim	21	10,032	Neg	Neg	Methyl-prednisolone	Alive
Gomez-Zorrilla S et al(14)	31	M	Astrocytoma	NA	Interstitial infiltrates	Yes SOB	No	Liver	Levetiracetam	46	NA	NA	NA	Dexa-methasone	Alive
O'Meara et al (15)	66	M	Hemochromat osis	NA	Interstitial infiltrates	Yes (SOB, dry cough)	No	Liver, Kidney Brain	Vancomycin	28	3620	Neg	NA	Hydro-cortisone	Alive
Aouam K et al (16)	14	M	Absence epilepsy	NA	Interstitial infiltrates	No	No	Liver	Lamotrigine, Carbamazepine	48	3450	Neg	Neg	Discontinue medication and supportive care	Alive
Lee JH et al(17)	29	F	Possible pulmonary tuberculosis	NA	Lobar infiltrate Pleural effusion	Yes (SOB)	No	Liver	Celecoxib, Ethambutol	38	4477	Neg	Pos	Methyl-prednisolone IVIG	Alive

Nawaz F et al(18)	19	M	Spinal fracture	NA	Pleural effusion, Interstitial infiltrates	Yes (SOB)	YES	Liver, Kidney	Titanium, Minocycline	45	8704	NA	NA	Prednisone	Alive
Yee BE et al(19)	18	F	HIV infection	NA	Lung nodules	No	YES	Liver	Raltegravir	35	1300	Ne	NA	Prednisone	Alive
Clayton BD et al (20)	26	M	Acne vulgaris	NA	Interstitial infiltrate ARDS	No	YES	Liver Brain	Minocycline	14	3250	NA	NA	IV corticosteroid	Alive
Bruwiere E et al (21)	35	F	Endocarditis	NA	ARDS	No	YES	Liver, Kidney	Teicoplanin, Moxifloxacin, Ciprofloxacin	9	590	NA	NA	Prednisone	Alive
Wilcox O et al (22)	39	M	DM, HLD	NA	ARDS	Yes (SOB)	YES	Kidney	Vancomycin	2	270	NA	NA	Methyl-prednisolone	Alive
Roca B et al(23)	22	F	Acne vulgaris	NA	ARDS	Yes (Cough, SOB)	YES	Liver	Minocycline	2	5760	Neg	NA	High dose steroids	Alive
Wang L et al(24)	50	M	Pulmonary tuberculosis	NA	Interstitial infiltrates Pleural effusion	No	No	Liver	Isoniazid, Rifampicin, Pyrazinamide	16	2870	NA	NA	Prednisone IVIG	Alive
Irga N et al(25)	4	F	Epilepsy	NA	ARDS	No	No	Liver Spleen	Carbamazepine	42	43,500	Neg	Pos	Methyl-prednisolone	Alive

Table legend: M = male, F = female, DM = diabetes, NA = Not applicable, HTN = hypertension, HLD = hyperlipidemia, CAD = coronary artery disease, PNA = pneumonia, ARDS = acute respiratory distress syndrome, SOB = shortness of breath, IVIG = Intravenous immunoglobulin, Neg = negative, Pos = positive
